# Optimal reduction of chemical oxygen demand and NH_3_–N from landfill leachate using a strongly resistant novel *Bacillus salmalaya* strain

**DOI:** 10.1186/s12896-017-0395-9

**Published:** 2017-11-28

**Authors:** Arezoo Dadrasnia, Mohd Sofian Azirun, Salmah Binti Ismail

**Affiliations:** 1 Institute of Biological Sciences, Faculty of Science, University of Malaya, 50603 Kuala Lumpur, Malaysia; 2 Institute of Research Management & Monitoring, University of Malaya, 50603 Kuala Lumpur, Malaysia

**Keywords:** Ammonia nitrogen, *Bacillus salmalaya*, Bioremoval, Bioengineering process, Landfill leachate

## Abstract

**Background:**

When the unavoidable waste generation is considered as damaging to our environment, it becomes crucial to develop a sustainable technology to remediate the pollutant source towards an environmental protection and safety. The development of a bioengineering technology for highly efficient pollutant removal is this regard. Given the high ammonia nitrogen content and chemical oxygen demand of landfill leachate, *Bacillus salmalaya* strain 139SI, a novel resident strain microbe that can survive in high ammonia nitrogen concentrations, was investigated for the bioremoval of ammonia nitrogen from landfill leachate. The treatability of landfill leachate was evaluated under different treatment parameters, such as temperature, inoculum dosage, and pH.

**Results:**

Results demonstrated that bioaugmentation with the novel strain can potentially improve the biodegradability of landfill leachate. *B*. *salmalaya* strain 139SI showed high potential to enhance biological treatment given its maximum NH_3_–N and COD removal efficiencies. The response surface plot pattern indicated that within 11 days and under optimum conditions (10% *v*/v inoculant, pH 6, and 35 °C), *B. salmalaya* strain139SI removed 78% of ammonia nitrogen. At the end of the study, biological and chemical oxygen demands remarkably decreased by 88% and 91.4%, respectively. Scanning electron microscopy images revealed that ammonia ions covered the cell surface of *B. salmalaya* strain139SI.

**Conclusions:**

Therefore, novel resistant *Bacillus salmalaya* strain139SI significantly reduces the chemical oxygen demand and NH_3_–N content of landfill leachate.

**Graphical abstract:**

Leachate treatment *by B. salmalaya* strain 139SI within 11 days.
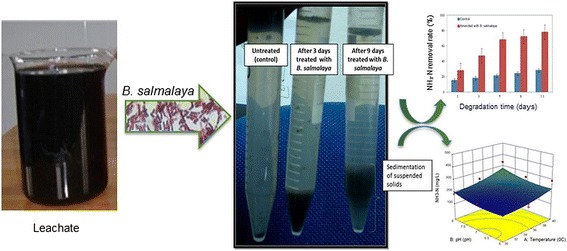

## Background

Over the past few years, the generation of municipal solid wastes in Malaysia has considerably increased due to rapid urbanization, capital development, and improving socioeconomic levels [1]. The amount of waste generated in Kuala Lumpur has rapidly increased from 587 tons/day in 1990 to 3701 tons/day in 2012 (0.9 kg/capita/day) [2]. In Malaysia, approximately 95% of collected wastes are dumped into 261 landfills given the inexpensive operating costs, easy set-up, and low supervision requirement of landfill sites [2, 3]. Consequently, the generation of landfill leachate is a major issue associated with the disposal of municipal solid waste. Inadequate treatment strategies for landfill leachate have recently become a considerable concern given that that the rate of waste generation is outpacing the rate of human population growth; furthermore, highly toxic hazardous waste is directly disposed into rivers or may percolate through soils and pollute receiving waters [3]. Various techniques have been proposed to address this environmental problem; among these techniques, bioaugmentation is an environmentally friendly and the most effective method to resolve the issue of landfill leachate [4, 5]. A serious issue of landfill leachate is its high content of ammonia nitrogen (NH_3_–N), which prevents the activity and growth of microorganisms due to accelerate eutrophication and an increased dissolved oxygen reduction as well as toxic effects on aquatic organisms. Conventional treatment methods are challenging, especially for mature or old landfill leachate with NH_3_–N levels [6–8]. Organic matter in leachate is quantified by biochemical oxygen demand (BOD) and chemical oxygen demand (COD).

Given their economy and feasibility, biological techniques are important methods for treating landfill leachate. Oxidation, electrocoagulation, microfiltration, and photo catalysis are commonly used to remove high COD and NH_3_–N from landfill leachate. All of these processes, however, are costly and can cause secondary pollution in some cases. Therefore, a relatively new treatment technique should be developed. Many techniques, such as nanofiltration [9], reverse osmosis [10], membrane bioreactor [11], up flow anaerobic sludge blanket combined with aerobic reactor [12], and adsorption by activated carbon [13], have been used to remove NH_3_–N and COD. Deng and Englehardt [14] treated leachate with hydrogen peroxide-improved iron and aeration; they found that the BOD/COD ratio increased from 0.02 to 0.17 and that NH_3_–N removal increased by 83%. These authors also reported that the electrochemical oxidation of landfill leachate under appropriate conditions removes most NH_3_–N and COD. However, the two main drawbacks of electro-oxidation are high energy consumption (more expensive operating costs than those of biological processes) and the potential formation of chlorinated organic compounds. Introducing microorganisms with high biodegradation ability and resistance to NH_3_–N, BOD, COD, and humic acid might be a promising solution to the problems associated with the treatment of landfill leachate. The presence of microbes in air, soil, and water environments enhances transformation and degradation during remediation [15]. Hence, microbes are important in the degradation of complex compounds. Bioaugmentation is a highly efficient and straightforward technology for removing pollutants from contaminated environments; this method could improve long-established treatment operations and decrease energy utilization [16, 17]. The main factor that affects the success of bioaugmentation is the ability of the added bacteria to adapt to and efficiently degrade pollutants under natural conditions [18, 19]. Highly adaptive bacteria stimulate the highly efficient removal of pollutant substances. To our knowledge, there are few studies on the removal of NH_3_–N from landfill leachate via bioaugmentation. The present study aimed to evaluate the efficiency of a novel bacteria (*Bacillus salmalaya* strain 139SI) that was isolated from agricultural soil in Malaysia; previous studies have shown that this bacterial strain has the ability and potential to degrade polycyclic aromatic hydrocarbons and sorb heavy metal [20, 21] and consequently decrease the high toxicity values of BOD, NH_3_–N, and COD in landfill leachate under different environmental circumstances.

## Methods

### Isolation and identification of strain

*B*. *salmalaya* was originally isolated from indigenous agricultural soil in Malaysia (2.99917°N, 101.70778°E). New strains were isolated with Brain–Heart–Infusion (BHI) agar as a highly nutritious growth medium which contains sodium chloride 5 g, disodium phosphate 2.5 g, dextrose 2 g, brain heart (infusion solids form) 8 g, agar 13.5 g, pancreatic digest of animal tissue 5 g, pancreatic digest of Casein 16 g, and supplemented with 5% sheep blood in 1 l. Among the isolated strains, 139SI exhibited strong hemolytic activity. On the basis of 16S rRNA sequencing and phylogenetic analysis, this strain was designated as a novel strain. Further information and molecular analysis of strain 139SI were reported recently [18, 19].

### Leachate sample

Leachate samples were obtained from an untreated leachate pond at the Bukit Beruntung landfill in Malaysia (3°25′32.14″N, 101°32′56.6″E); this landfill is a non-sanitary, open dumping and an operational landfill. Bukit Beruntung landfill (operated in 1992), which is visible from the North-South Highway, receives approximately 80 t of waste daily. The depression in the hilly area allows the expansion of the disposal site. Lack of enforcement by local municipality resulted in illegal dumping of municipal solid waste along the road to the landfill site.

### Determination of the minimum inhibitory concentration of NH_3_–N

To determine whether *B. salmalaya* strain 139SI is resistant to ammonia nitrogen, the following test was performed. First, strain pellets were prepared by centrifuging culture broth (OD_600_ = 1.0) at 3500 rpm for 15 min. These pellets were added into 10 mL of leachate with 1000 ppm concentration of NH_3_-N and incubated at 35 °C and 150 rpm for 72 h. Subsequently, bacteria were harvested and resuspended in leachate, which contained 2000 ppm ammonia nitrogen. The bacterial/leachate mixture was incubated under the above conditions. This process was repeated at different concentration and it was completed when the final concentration of NH_3_-N in the landfill leachate was 100 g/l. Approximately 20 μL of the each above mixture was spread onto a plate of solid medium and incubated at 35 °C for 16 h. The culture medium comprised BHI and leachate in 2:3 ratio and was solidified with agar. Pure colonies were selected, cultured, and stored for further experimental work.

### Bioaugmentation process

Pure *B. salmalaya* strain 139SI colony was cultured in BHI culture medium for 24 h at 33 °C and 150 rpm. Afterward, the biodegradation experiment was performed with 30 mL of landfill leachate inoculated with 3 mL bacterial strain (OD_600_ = 1) and incubated at 30 °C for 11 days at 150 rpm. Samples were collected and analyzed at 1, 3, 5, 8, and 11 days. To test the biodegradation capability of *B. salmalaya* strain 139SI and to remove the effect of indigenous bacteria, sterilized landfill leachate was degraded under same conditions.

### Effects of environmental conditions on bioaugmentation

To determine the effect of various conditions on the degradation efficiency of *B. salmalaya* strain 139SI, experiments were carried out at different temperatures (30 °C, 35 °C, and 40 °C), pH values (6, 7, and 8), and inoculum dosage (1, 3, and 5 mL). Experiments were performed in triplicate. Design-Expert Software was used to analyze experimental data. Significant effect on biodegradation was determined at 95% level (*P* < 0.05) using SPSS version 18.

### Elemental analysis

The decreases in the values of NH_3_–N, COD, and BOD were measured to evaluate the effectiveness of the microbial treatment of landfill leachate. COD concentration was determined on the basis of the agreed methods for Water and Wastewater examination (APHA 5220C). Samples were diluted and added to standard COD ampules and incubated in a dry incubator at 150 °C for 2 h. The COD tubes were allowed to cool down to room temperature and titrated with 0.05 M ferrous ammonium sulfate. NH_3_–N concentration was measured by APHA 4500 NH_3_ F-Phenate method. The sample was transferred to a 50-mL conical flask and mixed with the following solutions: phenol (1 mL), sodium nitroprusside (1 mL), and oxidizing solution (2.5 mL). The sample was covered and allowed to settle for at least 1 h and to develop color at room temperature (22–27 °C) in subdued light. Subsequently, the absorbance was read at 640 nm using spectroquant PHARO 100 [4]. BOD concentration was measured using APHA 5210 B method. Removal percentage was computed on the basis of the concentration measurement using the following equations:

1 $$ {\mathrm{Removal}}_{\mathrm{COD}}\left(\%\right)=\left[\left({\mathrm{COD}}_{\mathrm{i}}\hbox{--} {\mathrm{COD}}_{\mathrm{f}}\right)/{\mathrm{COD}}_{\mathrm{i}}\right]\times 100\%, $$

2 $$ {\mathrm{Removal}}_{\mathrm{N}\mathrm{H}3\hbox{--} \mathrm{N}}\left(\%\right)=\left[\left({\mathrm{N}\mathrm{H}}_3-{\mathrm{N}}_{\mathrm{i}}\hbox{--} {\mathrm{N}\mathrm{H}}_3-{\mathrm{N}}_{\mathrm{f}}\right)/{\mathrm{N}\mathrm{H}}_3-{\mathrm{N}}_{\mathrm{i}}\right]\times 100\%. $$

where *i* and *f* are the initial and final concentrations, respectively.

The morphology of cell surfaces (before and after adsorption) was evaluated with scanning electron microscopy (SEM, SEI quanta SEG 450, Netherlands).

## Results

### Raw leachate characteristics

The pH value of raw leachate was 8.3 ± 0.2. According to previous reports, the pH of old leachate is higher than 7.5; furthermore, the pH of stabilized leachate shows little variations and is in a fairly constant range of 7.5–9.0. The pH value of young leachate, however, is less than 6.5 [7, 22].

### Minimum inhibitory concentration of NH_3_–N

NH_3_-N tolerance test for the 139SI strain was performed in order to investigate the endurance ability of *Bacillus salmalaya* to growth at different concentration of NH_3_-N (from 1 to 100 g/l). However, none of the members of *Bacillus salmalaya* and has been reported to possess simultaneous COD and NH_3_-N removal abilities and such high NH_3_-N tolerance ability. Therefore, it was necessary to investigate the augmented performances of this novel bacteria to treat high NH_3_-N containing landfill leachate.

### Evaluation reduction of COD and NH_3_–N

**Fig. 1:**
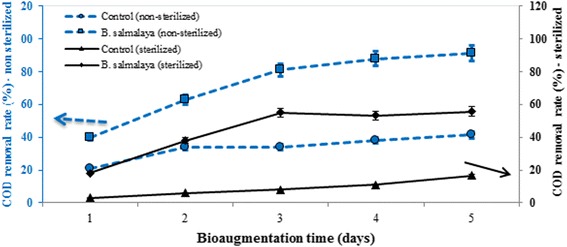
Amount of COD removed from unsterilized and sterilized landfill leachate by *Bacillus salmalaya* strain 139SI at different hydraulic retention times. *Vertical bars* indicate SE (*n* = 3)

**Fig. 2:**
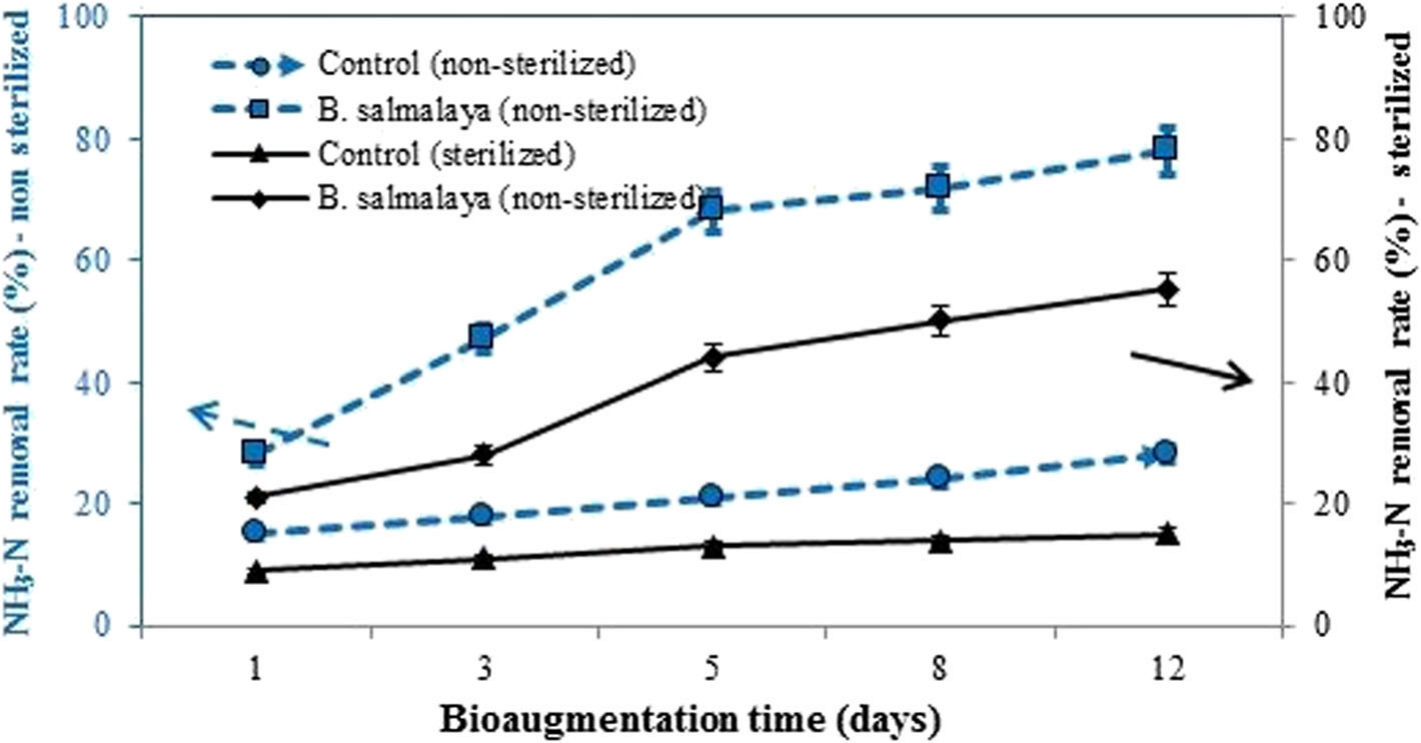
Percentage of NH_3_–N removed from unsterilized and sterilized landfill leachate by *B*. *salmalaya* strain 139SI at different hydraulic retention times. *Vertical bars* indicate SE (n = 3)

The successful bioaugmentation and removal of leachate depend on the ability of the introduced bacteria to perform its activities and survive in different environmental conditions. Figure 1 shows COD removal rate in unsterilized and sterilized landfill leachate during the 11 days of study. Statistical analysis showed a significant difference between leachates that were amended with culture or with the control treatment. The highest COD removal rate was observed in leachates that were amended with bacteria. The obtained results confirmed the ability of *B*. *salmalaya* strain 139SI to decrease COD under unsterilized conditions with a high rate of 81% after 5 days; this rate became constant in the following days and continued to increase and reached approximately 91.4% on day 11 (Fig. 1). The same trend was observed in sterilized amended samples, which showed a maximum COD reduction of 55.7% on day 11 (data showed no further increase in COD removal with the extension of degradation time). Sterilized landfill leachate without bacterial amendment was used as the control. The control showed 39% removal rate, which was lower than that of the inoculum. This result proved the significant effect of strain on degradation. NH_3_–N removal under both sterilized and nonsterilized conditions showed trends that were similar to those of COD removal, as shown in Fig. 2. The highest percentage of NH_3_–N reduction under unsterilized conditions was 78% in treatments that were amended with strain 139SI on day 11; this percentage was higher than that of the indigenous bacteria (28.1%).

### Correlations between BOD and COD

**Fig. 3:**
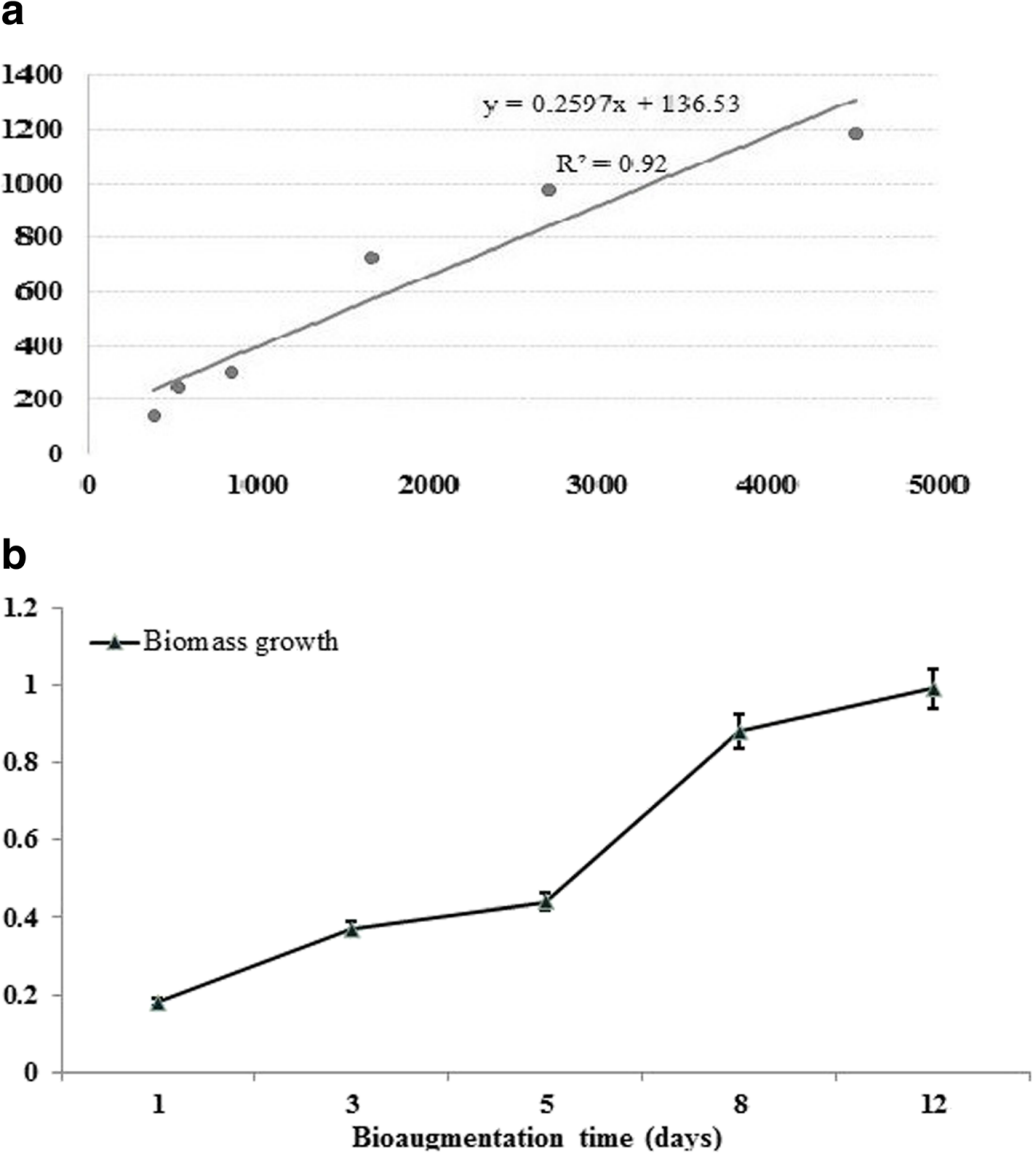
**a** Correlation between biochemical oxygen demand and COD. **b** Microbial growth kinetics. (Non-sterilized leachate sample) *Vertical bars* indicate SE (n = 3)

The high correlation (r^2^ = 0.92) between BOD and COD indicated the presence of organic matter in non-sterilized leachate landfill (Fig. 3a); organic matter can be readily degraded by the application of microorganisms. The organic matter in leachate was characterized on the basis of the different levels of biodegradability, which was defined with the mass concentration ratio of BOD: COD. This ratio indicates the degrees of both chemical and biological decompositions of pollutant leaching from landfills. The low BOD/COD ratio indicated the increased concentration of non-biodegradable organic compounds and consequently the difficulty of biodegradation [23, 24]. In the present study, this ratio continued to slowly and steadily increase from 0.26 to 0.36 with the increase of time treatment after microbial amendment. Biomass growth kinetics for non-sterilized sample amended with strain 139SI was observed during the 11 days of study (Fig. 3b). The high amount of biomass demonstrated that the *B*. *salmalaya* strain can tolerate the high COD and NH_3_–N concentrations and can enhance landfill leachate treatment.

### Response surface plots

**Fig. 4:**
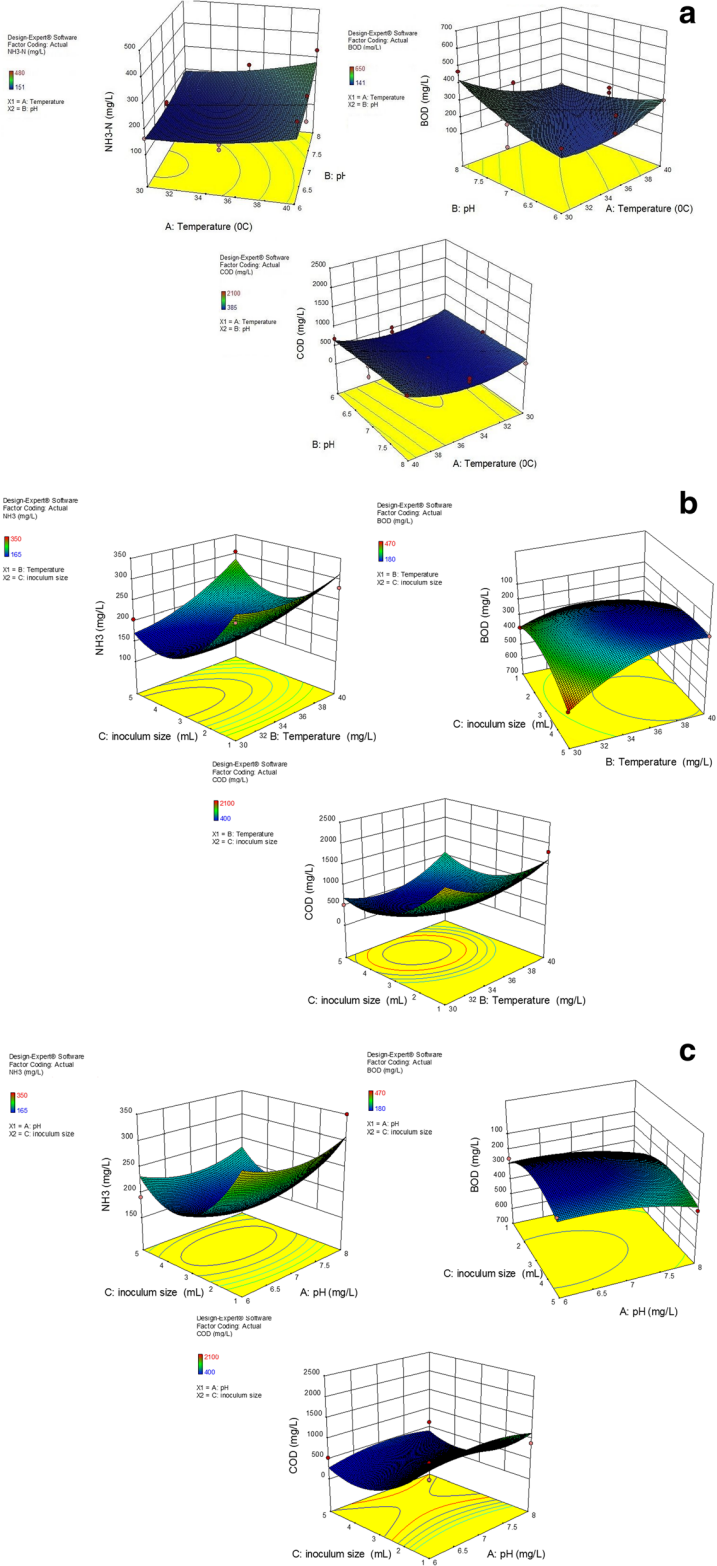
Influence of inoculum size, pH, and temperature on NH_3_–N, BOD, and COD removal

The effect of temperature (I), pH (II), and inoculum size (III) at three levels were investigated to optimize the removal of COD, BOD, and NH_3_–N by *B*. *salmalaya* strain 139SI. 3D response surfaces were drawn using Design-Expert Software 10. As shown in Fig. 4a, b, and c, the highest reduction in NH_3_–N (151 mg/L) occurred at pH 6 (II_1_), temperature of 35 °C (I_2_), and inoculum size of 3 mL (III_2_), which were identified as optimal conditions. According to statistical analysis (F values), the differential performance of variable parameters followed the order II > I > III. pH, temperature, and inoculum size exerted the largest effect on the removal rate of ammonia nitrogen (*P* value lower than 0.05).

### SEM analysis

**Fig. 5:**
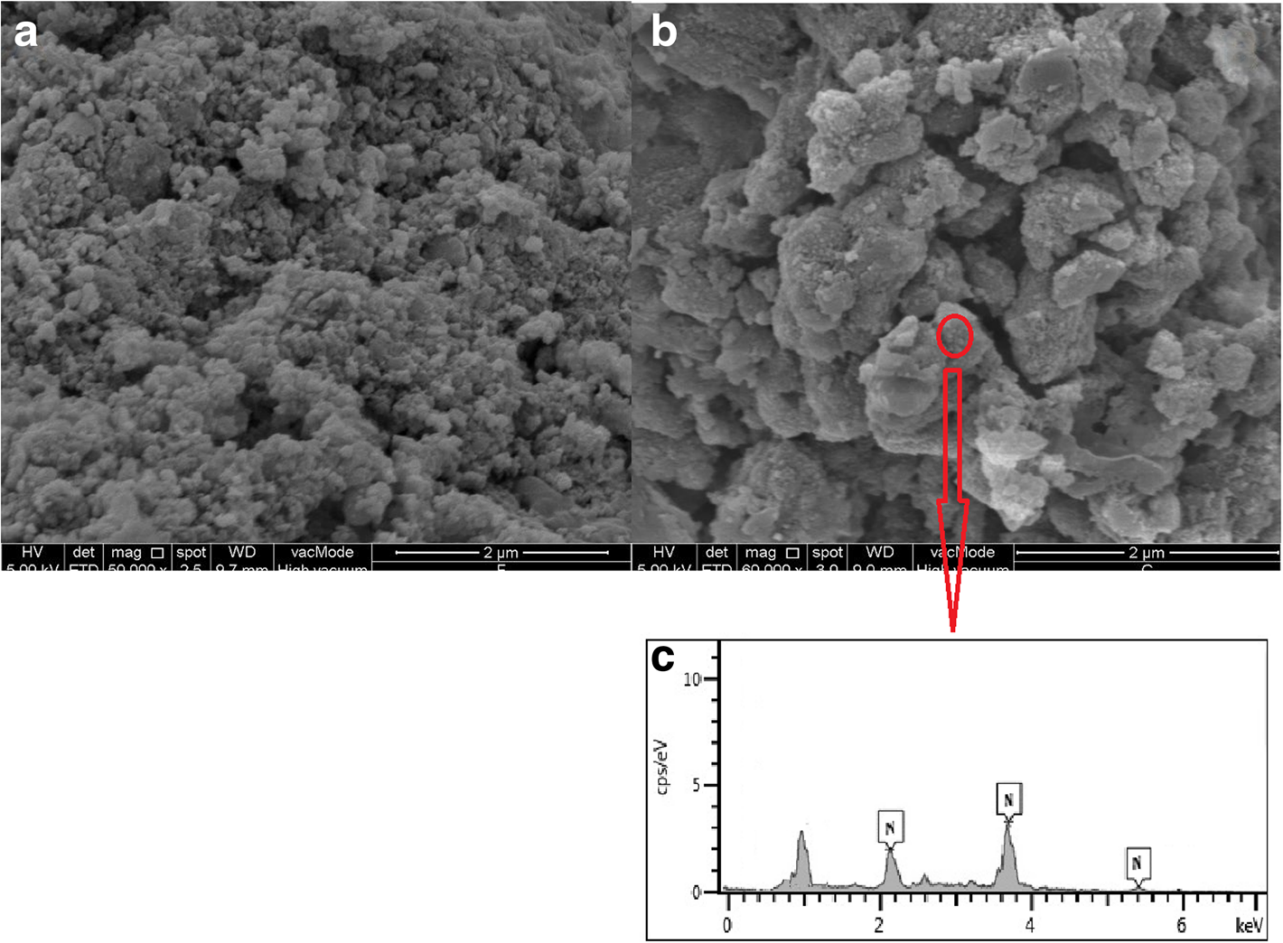
Scanning electron microscope micrographs **a** before, **b** after bioaugmentation, and **c** EDX after the bioaugmentation process

Surface characterization via SEM is a useful, simple qualitative method to demonstrate the microbe morphology in leachate [25]. The surface morphology of the cells was analyzed via SEM before (Fig. 5a) and after (Fig. 5b) use in bioaugmentation. Bacterial cells were thin and rod-shaped prior to the treatment and changed in morphology after use in biodegradation (Fig. 5b). This event would describe the mechanism of adsorption that is attributed to the coverage of the cell surface, which appeared large and spongy, with ammonia ions. EDX as a useful tool for evaluating the elemental and chemical analyses of biosorbents was illustrated in the Fig. 5c. The percentage of nitrogen ions were presented as final peaks and demonstrated that the ions were attached to the cell surface. The EDX only infer the N atom, the reason for such readout in EDX might be contributed to the present of protein on the cell surface, or cell lysate deposit.

## Discussion

In this study, BOD and COD amounts were 1189 ± 62.8 and 4517 ± 82.8 mg/L, respectively. These obtained values exceeded the authorized or permissible values issued by the Malaysian Environmental Quality Acts 127 and 1974 [26]. This result clearly showed that leachate required treatment to be environmentally acceptable. The BOD_5_/COD ratio is an indicator of the changes in the amount of biodegradable compounds in landfill leachate [27]. A BOD_5_/COD ratio of 0.26 indicates that the leachate is stable and poorly biodegradable [22, 28]. In this study, NH_3_–N concentration in the leachate was 685 ± 14.7 mg/L. The increasing amount of NH_3_–N with increasing landfill age likely results from the fermentation and hydrolysis of the nitrogenous fragments of biodegradable refuse [25, 29]. The results of the tolerance test proved the ability of *B. salmalaya* strain 139SI to survive under high NH_3_–N concentration as high as 70 g/l, as evidenced by the high cell counts after 16 h of culture. This result confirmed that our strain possessed high endurance under specific environmental pressures. The maximum COD reduction of non-sterilized leachate without culture addition was approximately 41.4%, which showed that the inoculum is a key factor in enhancing biodegradation. The higher removal rate in the treatment samples that were amended with strain 139SI than that in the control could be attributed to the effect of enzyme production, biomass activity, and growth efficiency of the microorganism.

The results showed that *B*. *salmalaya* strain 139SI had higher removal efficiency for COD than for NH_3_–N. Sterilized degradation was investigated to eliminate the effect of autochthonous bacteria and to assess the ability of 139SI in the bioaugmented treatment of landfill leachate. During the whole experiment, the COD reduction continued to increase in control treatments; the rate of increase, however, was not significantly evident compared with that of amendment treatments. Bioaugmentation displayed higher effectiveness in COD removal among all amended treatments. Removal efficiency of NH_3_–N remarkably increased after the third day (47%) and improved until the last day of study. In a similar study, [6] reported that under optimum conditions (30 °C, pH 7.33, 4.14 days, and 170 rpm), 94.7% of NH_4_^+^–N was removed from landfill leachates that were treated with 3.5 mL of inoculated domesticated bacteria. The same trend was obtained for the sterilized samples (Fig. 2). Amending the sterilized leachate with bacterial culture increased removal rates by 40% compared with the sterilized control without the strain. This result showed that almost all bacteria were killed due to sterilization (in this case, only 15% NH_3_–N could be removed). These results also showed that the degradation ability of *B*. *salmalaya* strain 139SI was in the order of COD and NH_3_–N. In our future study, we will propose a simple field trial to explore the potential of strain 139SI in landfill leachate treatment.

The highest reduction on COD (385 mg/L) and BOD (141 mg/L) was recorded at pH 7 (II_2_), inoculum size 3 mL (III_2_), and 30 °C (I_1_). These results indicated significant interactions between each factor during bioaugmentation. A recent research conducted by [25] on the Pulau Burung Landfill Site in Malaysia indicated that the optimum variable conditions for COD and NH_3_–N removal rates of 65.5% and 60.2%, respectively, under anaerobic condition were achieved with 100 mL of organism at pH 7 in a period of two weeks.

## Conclusions

Results demonstrated that bioaugmentation with the novel strain can potentially improve the biodegradability of landfill leachate. *B*. *salmalaya* strain 139SI showed high potential to enhance biological treatment given its maximum NH_3_–N and COD removal efficiencies. The maximum NH_3_–N removal of >65% and >75% was achieved after 5 and 11 days, respectively. COD (91.4%) and BOD (88%) considerably decreased after the study period. Therefore, this bacterial strain has potential low-cost applications in the treatment of wastewater with high COD, BOD, and NH_3_–N contents. Results verified that applying a single strain to landfill leachate, which contains many kinds of pollutants, is a fast and efficient method to remove high levels of NH_3_–N and COD. This study provides useful information for the design and management of landfill leachate for the realistic prediction of future trends.

## Abbreviations

BHI: Brain–Heart–Infusion
BOD: Biochemical oxygen demand
COD: Chemical oxygen demand
NH_3_–N: Ammonia nitrogen
OD: Optical density
SEM: Scanning electron microscopy
